# Resolvin D1 Reduces the Immunoinflammatory Response of the Rat Eye following Uveitis

**DOI:** 10.1155/2012/318621

**Published:** 2012-12-10

**Authors:** Rossi Settimio, Di Filippo Clara, Ferraraccio Franca, Simonelli Francesca, D'Amico Michele

**Affiliations:** ^1^Department of Ophthalmology, Second University of Naples, 80100 Naples, Italy; ^2^Department of Experimental Medicine, Section of Pharmacology “L. Donatelli”, Second University of Naples, Via Costantinopoli 16, 80138 Naples, Italy; ^3^Department of Clinical, Public and Preventive Medicine, Second University of Naples, 80138 Naples, Italy

## Abstract

This study investigated whether the administration of resolvin D1 to rats with endotoxininduced uveitis (EIU) ameliorates the immuno-inflammatory profile of the eye. 24 h after the administration of 200 **μ**g LPS into the footpad of Sprague-Dawley rats, severe changes of the structure of the eye occurred concomitantly with a severe inflammatory and immune response. These latter included strong infiltration of PMN leukocytes CD11b^+^ T-lymphocytes CD4^+^ and CD8^+^ within the eye and a significant release of the cytokines/chemokines TNF-alpha, CXCL8, and RANTES too. Bolus of resolvin D1 (RvD1; 10–100–1000 ng/kg in 200 **μ**L of sterile saline via the tail vein) significantly and dose-dependently (i) reduced the development of the ocular derangement caused by LPS; (ii) reduced the clinical score attributed to EIU; (iii) reduced the protein concentration and myeloperoxidase activity (MPO) in aqueous humor (AqH); and (iv) reduced neutrophils, T-lymphocytes, and cytokines within the eye.

## 1. Introduction 

Endotoxin-induced uveitis (EIU) is an animal model of acute ocular inflammation. Usually, this experimental pathology lasts up to 72 hours and has similarities with the human pathology [1]. Cytokines, chemokines, nitric oxide (NO), and impairment of stress-sensitive enzymes such as heme oxygenase-1 increase the inflammatory response to EIU. Augmentation of local recruitment of PMN promotes the inflammation and cell injury of the ciliary bodies in this pathology [1]. Thus, prospectively pharmacological agents that promote endogenous defensive responses, reduce the burden of inflammatory mediators released within the eye structures or reinforce the resolution of the inflammation that may exert cytoprotection and good outcome for uveitis. 

A growing body of evidence indicates that a good resolution of the inflammation could be achieved by means of resolvins. These are a family of potent lipid mediators derived that promote the resolution of the inflammatory response back to a noninflamed state [2]. Resolvin D1 (RvD1) is the major actor of the resolvins family; it is produced physiologically from the sequential oxygenation of DHA by 15- and 5-lipoxygenase [2] and it has effects on important components of the inflammation [3]. Resolvin D1 reduces human polymorphonuclear leukocyte (PMN) transendothelial migration, the earliest event in acute inflammation, and exhibits a dose-dependent reduction in leukocyte infiltration in a murine model of peritonitis with a maximal inhibition of ~35% at a 10–100 ng dose [4]. RvD1 also acts as a scavenger of cytokines and chemokines from the inflamed site and inhibits the production of PMN-derived free radicals [5–8]. Other biological actions have been reported, with a therapeutical potential such as a reduction in inflammatory pain [9]. 

On this base, we have investigated whether the stimulation of the resolutive phase of the inflammation through the use of the resolvin D1 ameliorates the immunoinflammatory profile of the rat eye following experimental uveitis. 

## 2. Materials and Methods

### 2.1. Induction of EIU

Male Sprague-Dawley rats (180–220 g) were injected in one footpad with 200 *μ*g of lipopolysaccharide (LPS, *Salmonella minnesota*, Sigma, St Louis, MO, USA) in 0.1 mL of sterile pyrogen-free saline [1] for the induction of EIU. The rats were treated with vehicle, LPS or LPS + resolvin D1 (RvD1) (*n* = 6 for each) and killed 24 h after treatment. The doses of RvD1 (10–100–1000 ng/kg) were chosen in the range of those used in murine models of inflammation [4]; they were injected by intravenous bolus (in 200 *μ*L of sterile saline) via the tail vein 1 h following LPS injection.

### 2.2. Clinical Score Attributed to EIU

Animals were examined with a biomicroscope 24 h after LPS injection. Clinical manifestations of EIU were graded from 0 to 4 in a blinded fashion according to the previously reported scoring system [10, 11]: 0 = no inflammatory reaction; 1 = discrete dilation of iris and conjunctival vessels; 2 = moderate dilation of iris and conjunctival vessels with moderate flare in the anterior chamber; 3 = intense iridal hyperemia with intense flare in the anterior chamber; and 4 = same clinical signs as 3 with presence of fibrinoid exudation in the pupillary area and miosis. No signs of uveitis were observed in the animals at the beginning of each experiment. Clinical EIU was considered positive when the score assigned was >1. EIU clinical data shown were representative of 3 sets of experiments and presented as mean ± SEM of 6 observations.

### 2.3. Myeloperoxidase Activity (MPO)

Immediately after the biomicroscope examination, the animals were killed with an overdose of anesthesia. Aqueous humor (AqH) was collected immediately from both eyes by an anterior chamber puncture (30–40 *μ*L/rat), using a 30-gauge needle under a surgical microscope and stored in ice water until testing. The MPO reaction was performed as previously described by Rossi et al. [1]. Data are reported as units of MPO activity. One unit of MPO activity has been reported to be equivalent to approximately 2 × 10^5^ PMN [12, 13].

### 2.4. Eye Samples

After 24 h of EIU, the eyes were harvested and cut in two halves. One half of each eye was immediately fixed by immersion in 10% buffered formalin and paraffin-embedded for immunohistochemistry. Sections were serially cut at 5 *μ*m, placed on lysine-coated slides, and stained with hematoxylin and eosin and with the trichrome method. The other half of each eye was immediately frozen in liquid nitrogen and stored at −80°C for the later biochemical assays described below. Subsequently the frozen tissues were homogenized in a solution containing 0.5% hexa-decyl-trimethyl-ammonium bromide dissolved in 10 mM potassium phosphate buffer (pH 7) and centrifuged for 30 min at 4,000 ×g at 4°C.

### 2.5. Immunohistochemistry

Paraffin-embebbed eye samples were treated with an xylene substitute (Hemo-De; Fisher Scientific) in order to remove the paraffin, and tissue sections were rehydrated with ethanol gradient washes. Tissue sections were quenched sequentially in 3% hydrogen peroxide aqueous solution and blocked with PBS 6% nonfat dry milk (Biorad, Milan, Italy) for 1 h at room temperature. Sections were then incubated with specific antibodies anti-CD11b, anti-CD4, and anti-CD8 (Santa Cruz Biotec, USA). Sections were washed with PBS and incubated with secondary antibodies. Specific labelling was detected with a biotin-conjugated goat anti-rabbit IgG and avidin-biotin peroxidase complex (DBA, Milan, Italy). The specimens were analyzed by an expert pathologist (intraobserver variability 6%) blinded to the experimental protocol. Six distinct preparations for each group of animals were done and 20 microscopic fields were analyzed in each preparation at 400x magnification. The total immunopositive particles were counted and expressed per total area. 

### 2.6. Cytokine Quantification in Tissue Homogenates

TNF-alpha levels in tissue homogenates (50 *μ*L) were determined using a commercially available ELISA specific for the rats cytokine, purchased from R&D Systems (Abingdon, UK). Briefly, tissue supernatant aliquots (50 *μ*L) were assayed for TNF-alpha and compared to a standard curve constructed with 0-1 ng/mL of the standard cytokine. The ELISA showed negligible (<1%) cross-reactivity with several murine cytokines and chemokines (data as furnished by manufacturer). A similar procedure was followed for determination of the chemokines CXCL8 and RANTES by ELISA (R&D Systems, UK) and used according to the manufacturer instructions. 

### 2.7. Statistical Analysis

All values are expressed as mean ± SEM of number (*n*) of rats for the *in vivo* experiments. Statistical analysis was assessed either by Student's *t*-test (when only two groups were compared) or one-way ANOVA followed by Dunnett's test (more than two experimental groups). A probability *P* value less than 0.05 was considered significant to reject the null hypothesis. 

## 3. Results

### 3.1. EIU Associated Clinical Manifestations and Tissue Damage

**Figure 1:**
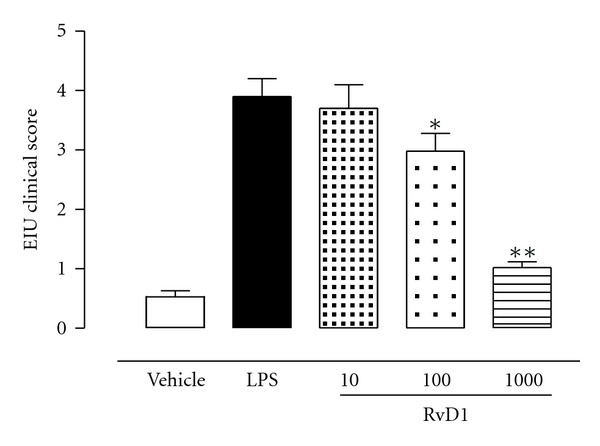
Effects of resolvin D1 on clinical development of EIU. The rats were treated with vehicle (PBS) and resolvin D1 (RvD1; 10, 100, 1000 ng/kg) 1 hour before LPS (200 *μ*g/rat) injection and were evaluated 24 h after LPS injection. Clinical manifestations of EIU were graded as reported in test (see Section 2). Values are reported as the mean ± SE, *n* = 6 per group. *P < 0.05 and **P < 0.01 compared with LPS-treated group.

24 h after the administration of 200 *μ*g LPS into the footpad of Sprague-Dawley rats, severe changes of the structure of the eye occurred with a clinical score of 3.90 ± 0.3 attributed (Figure 1). RvD1 (10–100–1000 ng/kg) dose-dependently attenuated the development of the ocular inflammation caused by LPS and improved the clinical score attributed in a dose-dependent manner (Figure 1). Particularly, the insurgence of EIU was effectively reduced (22.6 ± 1.4% less) by the intermediate dose of RvD1, while the highest dose protected the eye until the 74 ± 3% (Figure 1). The uveitis clinical scores for these two doses were significantly reduced to 1.02 ± 0.2 (*P* < 0.05) and 2.98 ± 0.3 (*P* < 0.01) compared with vehicle-treated group. The RvD1 lowest dose had no significant changes (Figure 1).

**Figure 2:**
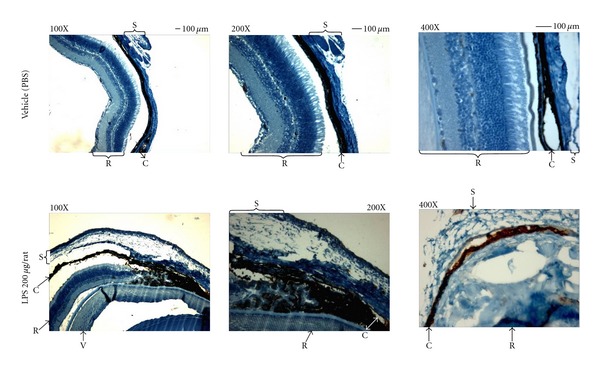
Sections showing representative immunohistochemistry of rats eye tissues treated with vehicle (PBS) and LPS (200 *μ*g/rat). Magnification was 100x, 200x, and 400x. C = choroid; Cb = ciliary body; R = retina; S = sclera.

**Figure 3:**
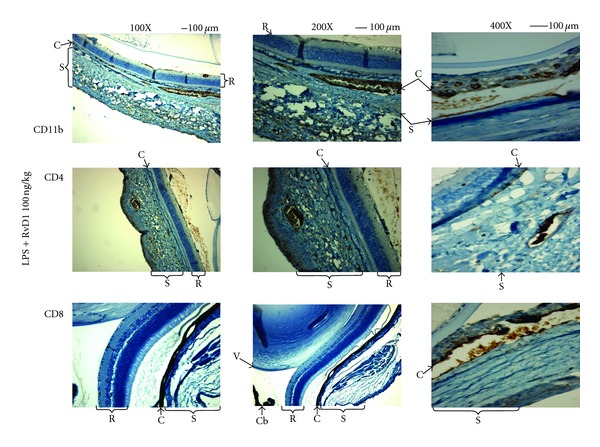
Representative immunohistochemistry of eye tissues showing that treatment with resolvin D1 (100 ng/kg, 1 h post-LPS) reduces inflammation and immunostaining for CD11b and CD4 and CD8. Magnification was 100x, 200x, and 400x. C = choroid; Cb = ciliary body; R = retina; S = sclera; V = vitreous.

**Figure 4:**
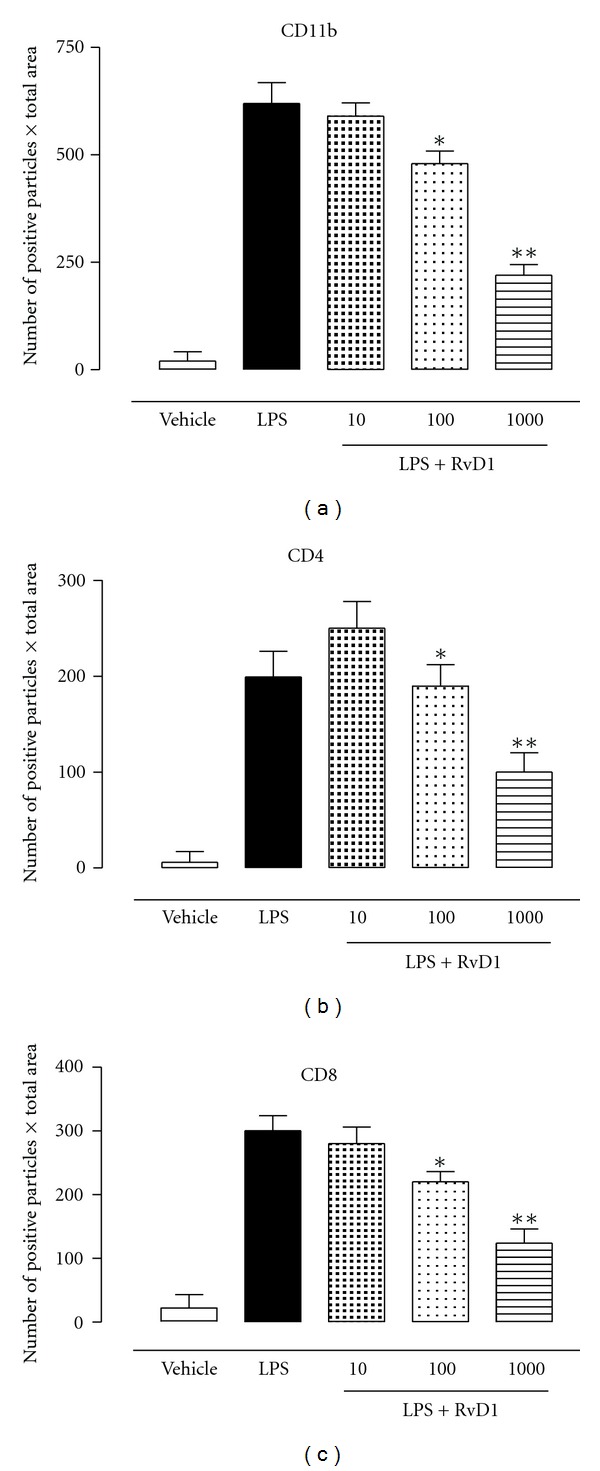
Graphs showing the number of positive particles per total area analyzed as described in Section 2. Data are obtained from 6 distinct preparations for each group of animals measuring 20 field of view for each preparation. Values are mean ± SEM (*n* = 6). *P < 0.01 versus LPS-treated group.

Concomitantly, a severe inflammatory and immune response rose within the eye of the rats. The immunohistochemistry performed on the eye revealed tissues were largely oedematous and telangiectatic with an oblong profile of the blood vessels (Figure 2), caused by the development of the oedema that tissues were largely oedematous and telangiectatic with an oblong profile of the blood vessels (Figure 2). Particularly, both the external fibrous (sclera) and vascular median tunics (choroid) were markedly infiltrated of flogistic elements (Figure 2). These were composed predominantly of PMN leukocytes CD11b^+^ and T-lymphocytes CD4^+^ and CD8^+^. In fact, Figure 3 showed that RvD1 reduced the number of infiltrated CD4^+^ and CD8^+^ particles within the perivascular tissue. At 24 h, the number of CD4^+^ particles for LPS + RvD1 treated animals were significantly lower than those for the LPS treated animals (*P* < 0.001) (Figures 3 and 4). LPS + RvD1 animals also had the lowest number of C8^+^ particles compared to the LPS group (Figures 3 and 4). The immunohistochemistry also showed that RvD1 reduced the infiltration of PMN leukocytes marked within the uvea. CD11b positive particles were mainly localized inside the blood vessels with no infiltration within the adjacent tissues; Figure 3 shows the effects of RvD1 100 ng/kg. The actions of RvD1 were evident for the doses of 100 and 1000 ng/kg, while 10 ng/kg gave no significant results in term of reduction of number of inflammatory components within the uvea (Figures 3 and 4).

### 3.2. Resolvin D1 Treatment and Biochemical Changes Associated with EIU

**Figure 5:**
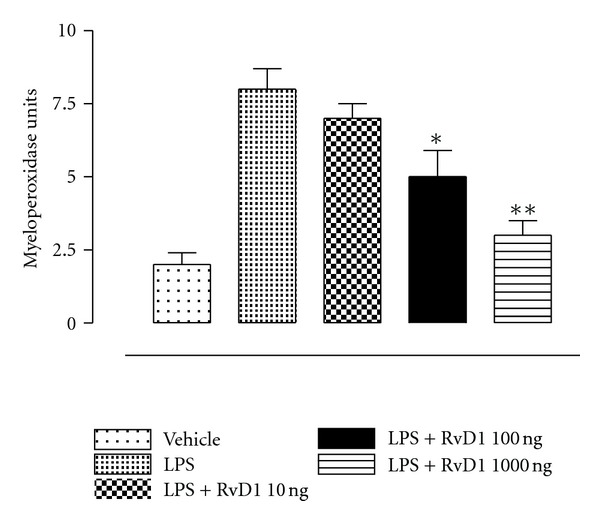
Effects of resolvin D1 treatment on MPO activity. The rats were treated with vehicle (PBS) and resolvin D1 (RvD1; 10, 100, 1000 ng/kg) 1 h after LPS (200 *μ*g/rat). Data are expressed as mean ± SE; *n* = 6 per group. *P < 0.05 and **P < 0.01 compared with LPS-treated group.

The development of EIU was paralleled by increase in MPO activity (Figure 5). Treatments of EIU rats with RvD1 1 hour after LPS caused a dose-dependent decrease of MPO activity in the AqH. The decreases significantly started from the dose of 100 ng/kg (*P* < 0.05 versus vehicle) and reached the maximum with the highest dose of 1000 ng/kg (*P* < 0.01 versus vehicle) (Figure 5). RvD1 10 ng/kg had no significant effect.

### 3.3. RvD1 Treatment on Cytokine and Chemokine Levels

**Figure 6:**
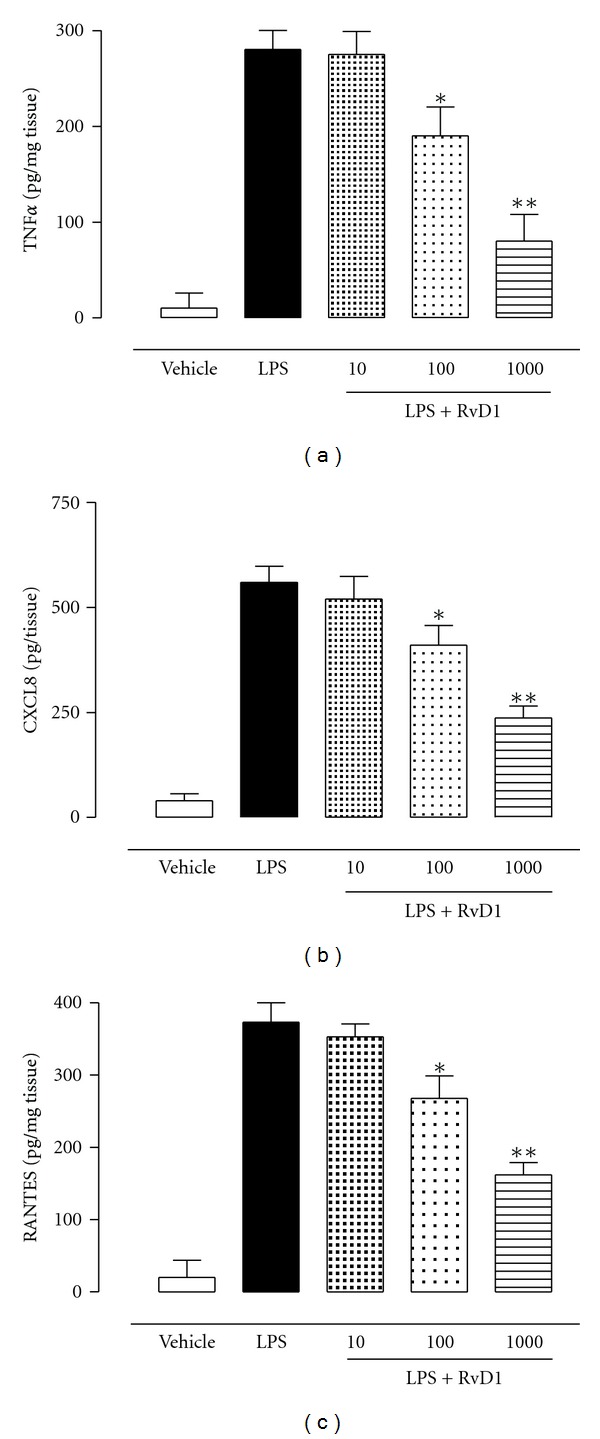
Levels of the cytokine and chemokines TNF-alpha, CXCL8, and RANTES within the homogenate of the uveal tissue. Data expressed as mean ± SE; *n* = 6 per group. *P < 0.05 and **P < 0.01 compared with LPS-treated group.

Tissue homogenates from the eyes of vehicle-treated rats had slightly appreciable levels of TNF-alpha, CXCL8, and RANTES (Figure 6). In contrast, tissue homogenates from EIU LPS-induced rats showed high levels of the TNF-alpha, CXCL8, and RANTES as 280 ± 20 pg/mg, 560 ± 38 pg/mg, and 373 ± 27 pg/mg, respectively (Figure 6). Resolvin D1 treatment, 1 hour after LPS, dose-dependently produced a significant reduction in either the cytokine and the chemokine levels within eye tissues (Figure 6).

## 4. Discussion

Here we report that stimulation of resolvin D1 pathway in rats undergoing experimental uveitis ameliorates the immuno-inflammatory profile of the external and median tunics of the eye, accounting for eye protection. 

Inflamed eye is the result of the altered functions of endothelial cells, leukocytes, retinal pigment epithelium, retinal neurons, glial cells, and other types of cells locally present. These cells are targets of signalling molecules such as lipid mediators and cytokines that favour the shifting of the tissue from a physiological shape to pathological one. Targeting one, or more than one, of these mediators with specific agents prevents inflammation or promotes resolution of it [14].

Resolvins are a class of endogenous molecules aimed to the resolution of inflammation. This latter response is limited at the site of the noxious stimulus, in order to restore the right homeostasis through specialized proresolving mediators with tissue-protective and resolution-stimulating functions [3]. They are biosynthesized from eicosapentaenoic acid and from docosahexaenoic acid and so are denoted as E series and D series, respectively [2]. Actually, resolvins are also formed from cycloxygenase-2 following the aspirin action. The actions of the resolvins include the scavenging of cytokines and chemokines from the inflamed site, the inhibition of the *de novo* production of cytokines and chemokines, the inhibition of the leukocytes trafficking/infiltration to inflamed tissue, and the inhibition of the production of PMN-derived free radicals [5–8]. However, an action on the recruitment of nonphlogistic monocytes and phagocytosis is also accredited [9]. 

From the molecular point of view, the proresolving properties of resolvins are exerted through the share of G-protein-coupled seven-transmembrane receptors located on human leukocytes with the anti-inflammatory peptide annexin 1 and chemerin. Receptors are called ALX/FPR2 (LXA_4_ receptor) and GPR32 (G-protein-coupled receptor) [15–18] and are aimed to translate the RvD1 signal into leukocytes activation and movement impediment. According to this evidence here we report that the pro-resolving properties of resolvin D1 is exerted on the typical actors leading experimental uveitis, the white blood cells recruited into the eye specimens. MPO, a sensible marker of leucocytes infiltration, and tissue immunoreactivity for the CD11b were remarkably reduced by the RvD1. PMN leukocytes activation and infiltration are the key events of inflamed eye [19], because PMN leukocytes adhere to, roll along, infiltrate the endothelial wall of blood vessels and release reactive oxygen species to the site attacked causing inflammation. Furthermore, this treatment also reduced the local generation of cytokines and chemokines which are known to promote leukocyte-endothelium interaction [20] and finally eye damage. Indeed, a cytokine able to increase the adhesive properties of the endothelial wall [21, 22], and that it is implicated in the pathology associated with experimental uveitis [23], the TNF-alpha was drastically reduced following the treatment of the rats with RvD1 as well as the chemokine CXCL8, a chemokine able to recruit neutrophils in rodent species during experimental inflammation [21, 24]. As chemoattractants, CXCL8 stimulates directional leukocyte migration and activates the expression of integrin on leukocytes such as CD11b, which increases leukocyte binding to the ligands ICAM-1 and VCAM-1 on the endothelium [19]. Taken together, the present results show that the persistent inflammation of the eye and tissue damage following uveitis could be controlled by means of RvD1. An intriguing hypothesis would be that the RvD1, acts through its receptor ALX/FPR2, ubiquitously present within the eye structures [25]. Consistent with this contention are the recent studies by Odusanwo et al. [26], in other settings. 

The resolution of the inflammation seems not be the only target to reach during uveitis; unfortunately, the immune response has a major responsibility in it and thus needs to be suppressed. Much experience on the immune-mediated damage has arisen from clinical and experimental models of uveitis [27–29]. However, the interplay between innate and adaptive immunity and the crossover between autoinflammatory and autoimmune conditions need relative exploration. Here we show that although the predominant infiltrating cell type in EIU is the PMN neutrophil, CD4^+^ and CD8^+^ T cells are also partners of this company. These cells have been found markedly infiltrated within the uveal structures together with PMN leukocytes. Over the last two decades, the understanding of immunopathogenetic mechanisms associated with EIU is increased and supports a fundamental role of T cells in it, especially CD4^+^ T cells. This is in accordance with a number of studies done through the last two decades supporting the fundamental role for T cells, especially CD4^+^ T cells [30]. Other studies showed that that anti-CD4 antibody significantly reduced the severity of EIU in endotoxin-responsive strains of mice, while an anti-CD8 antibody had no influence on the disease [31]. In 2002, Avunduk et al. [32] also showed CD4^+^ and CD8^+^ cells infiltration in the anterior uveal tract, paving the way to the later concept that activated lymphocytes can invade vascular endothelium by degrading subendothelial matrix, and so memory T lymphocytes may be the predominant introducers of the extra vascular tissues. Activated T lymphocytes selectively cross the blood-aqueous barrier, accumulate in the uveal tissue of EIU animals, and secrete a number of damaging elements [33]. 

RvD1 seems a good piece for this puzzle. RvD1 quantitatively reduced the number of infiltrated CD4^+^ and CD8^+^ elements within the perivascular uveal tissue. Also, RvD1 associated a reduction of the T-chemokine RANTES (Regulated on Activation, Normal T Expressed and Secreted) within the uveal tissues. RANTES, also known as CCL5, is an 8 kDa protein member of the interleukin-8 superfamily of cytokines. Firstly identified after T-cells activation [34], it was subsequently determined to be a CC chemokine and expressed in more than 100 human diseases. RANTES expression in T lymphocytes is regulated by Kruppel like factor 13 (KLF13) [35–37] and is chemotactic for T cells, eosinophils, basophils, neutrophils, and macrophages [38–40], and through its receptor CCR5 plays an active role in recruiting leukocytes into inflammatory sites [41]. RANTES, along with the related chemokines MIP-1alpha and MIP-1beta, has been identified as a natural factor secreted by activated CD8^+^ T cells and other immune cells [42]. With the help of particular cytokines (i.e., IL-2 and IFN-*γ*) that are released by T cells, CCL5 also induces the proliferation and activation of certain natural-killer (NK) cells to form CHAK (CC-chemokine-activated killer) cells [43]. It is also an HIV-suppressive factor released from CD8^+^ T cells [42]. Therefore, limiting the infiltration of immune cells into inflamed sites or reducing their products could be mandatory for return to eye homeostasis. 

In conclusion, our study shows that stimulation of the resolving phase of EIU through resolvin D1 pathway ameliorates the immuno-inflammatory profile of the rat eye.
